# Author Correction: Reflection from a free carrier front via an intraband indirect photonic transition

**DOI:** 10.1038/s41467-018-05836-8

**Published:** 2018-08-20

**Authors:** Mahmoud A. Gaafar, Dirk Jalas, Liam O’Faolain, Juntao Li, Thomas F. Krauss, Alexander Yu. Petrov, Manfred Eich

**Affiliations:** 1 Institute of Optical and Electronic Materials, Hamburg University of Technology, Hamburg 21073, Germany; 2 Department of Physics, Faculty of Science, Menoufia University, Menoufia 32511, Egypt; 3 SUPA, School of Physics and Astronomy, University of St. Andrews, St. Andrews, Fife KY16 9SS, UK; 4 Tyndall National Institute, Lee Maltings Complex, Dyke Parade, Cork T12 R5CP, Ireland; 5 Centre for Advanced Photonics and Process Analysis, Cork Institute of Technology, Cork T12 P928, Ireland; 6 State Key Laboratory of Optoelectronic Materials & Technology, Sun Yat-sen University, Guangzhou 510275, China; 7 Department of Physics, University of York, York YO105DD, UK; 8 ITMO University, 49 Kronverkskii Ave., 197101 St. Petersburg, Russia; 9 Institute of Materials Research, Helmholtz-Zentrum Geesthacht, Max-Planck-Strasse 1, Geesthacht D-21502, Germany

**Correction to**: *Nature Communications* 10.1038/s41467-018-03862-0; published online 13 April 2018

The original version of this Article contained an error in first sentence of the Acknowledgements, which incorrectly read ‘M.A.G, D.J., A.Y.P. and M.E. acknowledge the support of the German Research Foundation under grant no. EI 391/13-2, and appreciate the support of CST, Darmstadt, Germany, with their Microwave Studio Software.’ The correct version states ‘261759120’ in place of ‘EI 391/13-2’. This has been corrected in both the PDF and HTML versions of the Article.

