# Supplementary material for: Reflection from a free carrier front via an intraband indirect photonic transition
Source: Nat Commun. 2018 Apr 13;9:1447. doi: 10.1038/s41467-018-03862-0 (PMC5899136; doi:10.1038/s41467-018-03862-0)
Supplement: Supplementary file 1 — Supplementary Information [file 41467_2018_3862_MOESM1_ESM.pdf]

# Supplementary Information of "Reflection from a free carrier front via an intraband indirect photonic transition"

Gaafar et al.

**Supplementary Note 1: Structure of the slow light silicon PhC waveguide and measurement setup**

The employed single line defect PhC waveguide has been fabricated on a Silicon-on-Insulator substrate with slab height of 220nm. The length of the waveguide is 396 µm and is connected to the edges of the chip by polymer waveguides to reduce the coupling losses which are converted into silicon access waveguides by inverted tapers. The silicon access waveguides have a cross section of 3 × 0.22 $\mu m^2$ and a length of 5 mm, which are tapered down to a width of 0.85 µm at the input of the PhC over a length of 200 µm. In this study, the lattice constant of the PhC is 404 nm and the air-holes diameter is 240 nm. In order to achieve a waveguide with required dispersion, the first row of holes directly adjacent to the waveguide has been shifted 50 nm away from the waveguide center [1]. The oxide underneath the waveguide has been removed, in order to achieve a symmetric air cladding [1,2]. Coupling into the structure is carried out in an end fire configuration, with coupling losses of approximately -4dB per facet. The group velocity was measured using an external Mach-Zenhder interferometer [3].

The experiment is arranged as displayed in Supplementary Figure 1. A mode-locked fiber laser (Menlo system) with a 100 MHz repetition rate delivers pulses of approx. 100 fs duration with a wide spectrum ranging from 1500nm–1600nm. The output is connected to a grating filter with adjustable bandwidth and center frequency followed by an erbium-doped fiber amplifier (EDFA). Pulses with tunable center wavelength between 1525 nm –1590 nm and bandwidth between 0.1 nm – 4 nm (33 ps – 2 ps) can be produced from this configuration. The probe light is derived from a tunable diode laser (Photonetics PRI). It delivers up to 8 mW of CW light, tunable from 1500 to 1600 nm. The adjustment of optical power and polarization is carried out with a combination of half-wave plates and polarizers. The pump pulse is subsequently combined with the probe light through a 50/50 beam splitter.

The optical spectra are measured by the optical spectrum analyzer. To decrease the noise level the spectrum is scanned with 1 nm bandwidth.

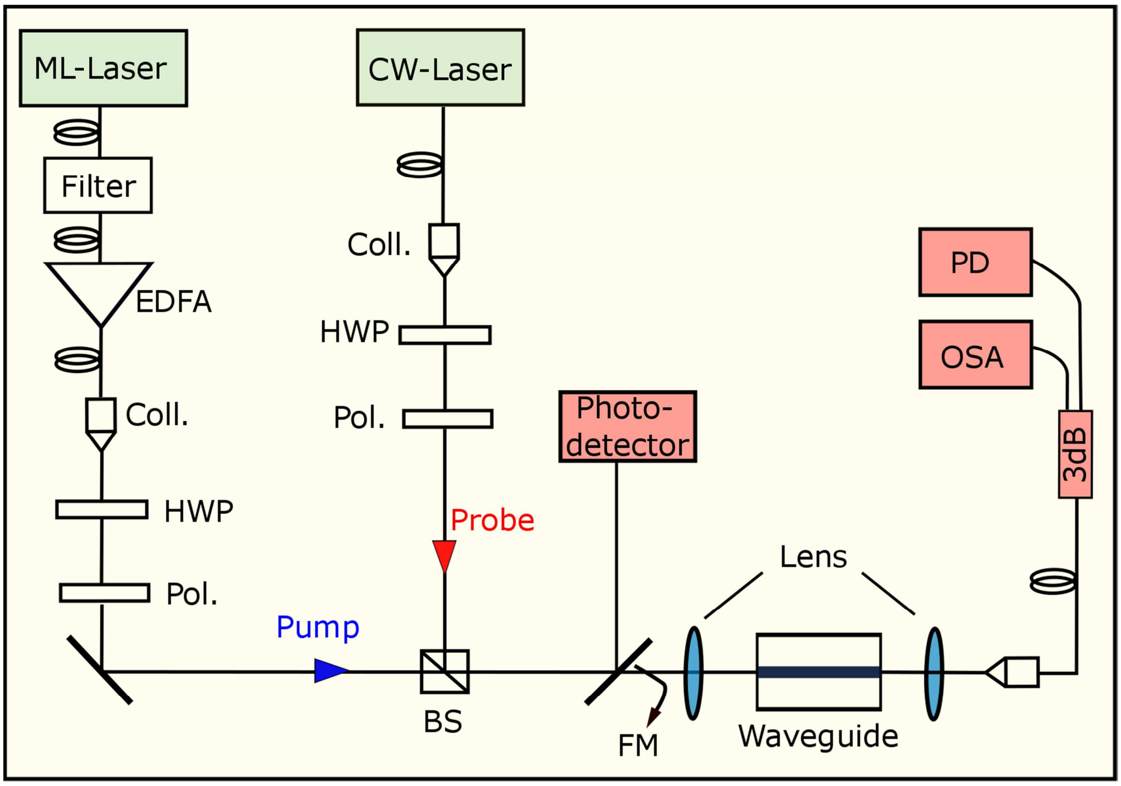


Supplementary Figure 1: Schematic of the experimental setup. EDFA, erbium-doped fiber amplifier; Coll., collimator; Pol., polarizer; HWP, half-wave plate; BS, beam splitter; FM, Flipping mirror; OSA, optical spectrum analyzer; and PD, photodiode.

## Supplementary Note 2: Free carrier generation and index change of an optical mode

The time dependence of the free carrier (FC) density $N(t)$ at a fixed point in space generated by two-photon absorption (TPA) of a pump pulse propagating in a slow light silicon waveguide is described by[4]:

$$\frac{dN(t)}{dt} = \frac{\beta}{2hf}\left(\frac{n_g}{n_{si}}\right)^2 I^2(t) - \frac{N(t)}{\tau_{FC}} \tag{1}$$

The first term on the right-hand-side of this equation describes the generation of free carriers via the TPA process, while the second term describe the decay of the generated free carriers,

characterized by the lifetime$\tau_{FC}$. Here, $h$ is Planck's constant, $\beta = 0.7\ cm/GW$ is the TPA coefficient in silicon at a frequency $f = 193.4\ THz$[5], $n_g$ is the pump group index, $n_{si}$ is the silicon refractive index, and $I(t)$ is the pump pulse intensity.

The FC induced refractive index change can be described by the empirical relation presented by Soref et al.[6]:

$$\Delta n(t) = -8.8 \cdot 10^{-22} N(t) - 8.5 \cdot 10^{-18} N(t)^{0.8} \tag{2}$$

We solve equation (1) numerically assuming a Gaussian pulse intensity $I(t) = I_0 \exp\left(-4 \ln 2\, t^2 / t_{\mathrm{pulse}}^2\right)$ with FC life time $\tau_{FC} = 1\,\mathrm{ns}$. The maximum of the generated FC density is given by:

$$N_{FC,\max} = \frac{\beta}{2hf} \left( \frac{n_g}{n_{si}} \right)^2 I_0^2 \sqrt{\pi} \frac{t_{pulse}}{\sqrt{8 \ln 2}} \tag{3}$$

When a small perturbation of the refractive index is applied, the frequency of an eigenmode will change according to[7]:

$$\Delta\omega = -\frac{\omega}{2} \frac{\int \Delta\varepsilon(r) |E(r)|^2 dr}{\int \varepsilon(r) |E(r)|^2 dr} \tag{4}$$

For homogeneous spatial distributions for $\Delta\varepsilon$ and $\varepsilon$ one gets from equation (4) the relation:

$$\frac{\Delta\omega}{\omega} = -\frac{\Delta n}{n} \tag{5}$$

Considering the dynamic switching configuration in our PhC waveguide, special attention should be paid to carrier diffusion in silicon. The spatial distribution of $\Delta n$ is restricted to the center region of the waveguide up to the surrounding first row of holes, which inhibits carrier diffusion out of the center region of the waveguide[8]. Assuming a small $\Delta n$ which is constant over the center region and zero elsewhere, compared to a spatially homogenous change of the

refractive index, this assumption will not lead to a considerable change of the slope of the shifted band diagram, and one gets from equation (4)[7]:

$$\frac{\Delta\omega}{\omega} = -\frac{\Delta n}{n} \cdot \text{(fraction of } \int \varepsilon |E|^2 \text{ in the perturbed regions)} \quad (6)$$

From this equation we can estimate the expected frequency shift of the optical mode according to the maximum refractive index change $\Delta n$ . For the presented slow light waveguide the fraction is close to one.

For $t_{pulse} = 6\,ps$, $n_g = 30$, a pulse peak power of 4 W at the input of the slow light waveguide, and by taking into account an effective mode area of the pump pulse of $0.5\mu m^2$ in the slow light waveguide, we estimate the maximum index change at the input of the waveguide of $\approx -3 \cdot 10^{-3}$, which corresponds to the generated FC density of $\approx 7 \cdot 10^{17}/cm^3$.

**Supplementary Note 3: Pump power decay**

We should mention that the pump power decays in both the access as well as the slow light waveguides due to absorption and scattering. We approximate the losses due FC absorption and TPA in the access waveguide by ≈ 2dB and neglect linear losses in this waveguides. This in turn will lead to a pump peak power of 4 W at the input of the slow light waveguide. We estimate total losses of ≈ 4 dB inside our slow light waveguide as a result of ≈ 2dB linear losses [9] and ≈ 2dB nonlinear losses due to FC absorption and TPA [10]. As the FC generation rate via TPA is proportional to local pump power squared, therefore the index front has 8dB exponential decay during propagation inside the waveguide.

**Supplementary Note 4: The intraband transition is not reversible**

The FC plasma mirror is asymmetric in its reflection properties. Due to the long lifetime, the FC front does not cause an opposite slope of the refractive index front at its rear side. Thus, the fast probe wave packet approaching the front from the rear side is transmitted through the

front and is blue shifted again. This is an interband transition, again, however, this time, the starting point originates from the switched band, then the probe wave packet is guided along the phase continuity line and finally lands at a higher frequency on the unswitched band. Supplementary Figure 2 shows the experimental output spectra recorded with and without pump pulses of an input probe at a wavelength of 1528.5 nm. Remarkably, with the pump pulses present, the induced indirect transition again leads to a blue shift of the probe frequency.

In contrast to the case presented in Fig. 3(a) of the main manuscript where the probe wave packet was travelling slower than the front, now the probe wave packet is travelling faster than the front. Therefore the probe wave already enters the waveguide in its switched zone, thus the initial state of the probe wave $(\omega_1, k_1)$ already lies on the switched band corresponding to $n_{si} + \Delta n_{FC}$. The final state of the probe $(\omega_2, k_2)$ is determined graphically from the crossing point of the phase continuity line and the solid band, as schematically illustrated at the inset of Supplementary Figure 2, which indicates that the final state of the probe lies on the band corresponding to $n_{si}$.

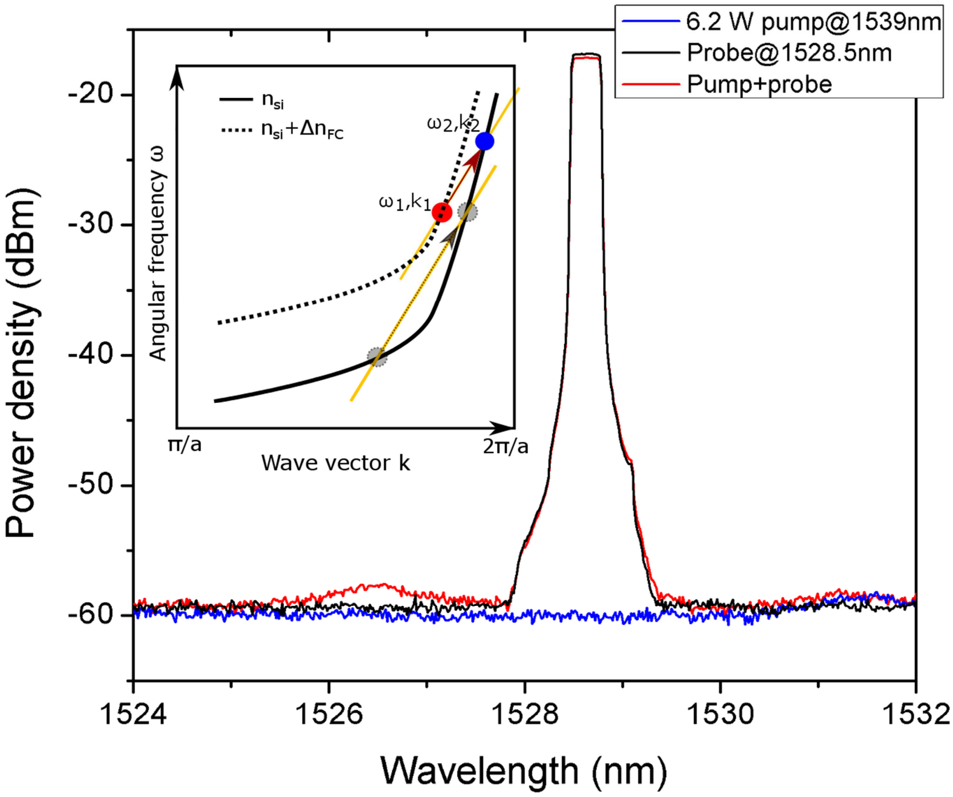


Supplementary Figure 2: Experimental spectra recorded at the output of our slow light silicon PhC waveguide of an input probe at a wavelength of 1528.5 nm and pump peak power of 6.2 W. The spectrum of the CW probe that has not interacted with the pump pulses (black trace), of the pump pulses alone (blue trace), and of both the pump pulses and the probe (red trace). Inset shows schematically the corresponding transition (red and blue dots). Grey dots represent the intraband transition when the faster pump pulse approaches the initially slower probe wave packet in the unswitched zone.

## Supplementary Note 5: Original calculated band diagram

Supplementary Figure 3 shows the original calculated dispersion band, without correcting the wave vector by the slope of the pump group velocity, as shown in the main manuscript. The solid curve represents the dispersion band of waveguide mode with refractive index $n_{\mathrm{si}}$, while the dashed curve indicates the waveguide mode with refractive index change of $-3{\cdot}10^{-3}$. Color dots indicate the locations of the input wavelengths of probe (red dot), pump pulse (orange dot), and the expected wavelength of the shifted probe wave (blue dot).

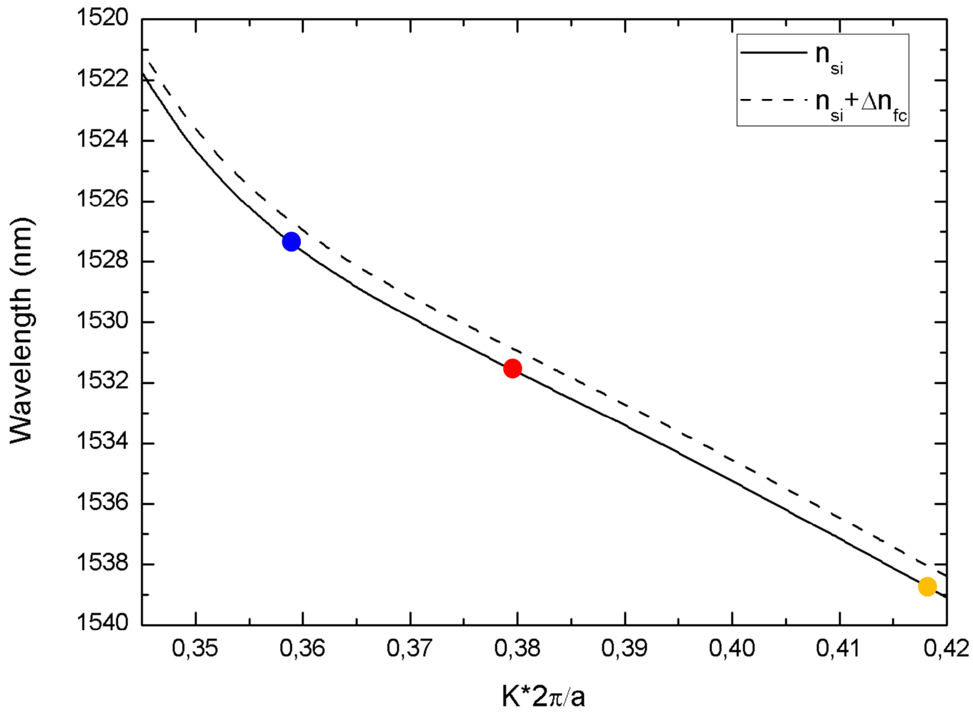


Supplementary Figure 3: The calculated dispersion band from the measured group index of our PhC. The solid curve represents the dispersion band of waveguide mode with refractive index $n_{si}$, while the dashed curve indicates the waveguide mode with refractive index change of -3·10$^{-3}$. Color dots indicate the locations of the input wavelengths of probe (red dot), pump pulse (orange dot), and the expected wavelength of the shifted probe wave (blue dot).

**Supplementary Note 6: Simulation of CW wave packets trajectories**

Here, we simulate the FC front and CW probe wave packets interaction inside the 400 $\mu m$ long waveguide. We focus on the part of the incident probe light that interacts with the front and ignore both the unperturbed input CW probe light as well as the components that are slightly down shifted in frequency due to slow FC decay after the front propagation. We assume that we can split the CW probe into wave packets and track their trajectory and frequency shift. At the end of the waveguide we calculate the energy of the shifted probe by adding up the energy of the shifted wave packets. This approach neglects the spectral width of the wave packet and interference effects between the wave packets and thus represents a ray optics approximation.

In our simulation we use a front with a linear slope. We took into account the FC absorption of the probe scaling linearly with FC concentration. The absorption coefficient is 30 $cm^{-1}$ at FC concentration of $7 \cdot 10^{17}/cm^3$ [11], taking a slow-down factor of 10 into account. Also, we consider an exponential decay of the front refractive index change during propagation inside the waveguide due to scattering and TPA and FC absorption with decay constant corresponding to 8dB decay in 400μm waveguide. We start with a defined frequency of the wave packet and calculate its wavenumber and group velocity at the input in accordance to its position in respect to the front. In a small time step a wave packet propagates a small distance according to its group velocity. Being inside the front the wave packet accumulates a frequency shift according to eq. (1) in the main manuscript, a wavenumber shift according to $\Delta k = \Delta\omega/v_f$ and is subject to absorption according to the local FC concentration. This leads to a change of the group velocity which determines how the wave packet further propagates. Thus, we track the signal wave packets in space and time and accumulate the frequency shift. At the end at the output of the waveguide we obtain the wave packets with their new frequency and smaller energy due to FC absorption.

Supplementary Figure 4 presents the simulation results for an input probe light at 1531.5 nm with 6 μW CW output power at two different values of $\Delta\omega_{shift}$. Here, we chose the front rising time of 6 ps and front group index of 30. The time step for evaluation of the new position and new group velocity of the wave packet in the simulation is 0.1 ps. The starting time of the first wave packet is 5 ps ahead of the starting time of the front, while the starting time of the last wave packet is 7 ps after the front has started. Here, the number of simulated wave packets is 120. In Supplementary Figures 4 (b), and (d), we show the trajectories of only few wave packets for clarity. Black lines represent those wave packets that interacting with the front and undergo interband and intraband transitions. The two magenta lines illustrate the extent of the index front with a slope equal to the group velocity of the pump and with a rising

time of 6 ps. The 40 ps simulation time is associated with time spent by the front with a group index of 30 inside the 400 μm long waveguide.

Supplementary Figures 4 (a), and (b) shows the calculated output average power of the wave packets integrated over frequency intervals of 1 nm and their trajectories for the case of $\Delta\omega_{shift}$ being insufficient to induce intraband transitions. We can see that most of the wave packets energies are concentrated in the frequency interval corresponding to interband transitions ( ≈ 1nm blue shift from the input wavelength at 1531.5 nm). We can see also from the trajectories of the wave packets that their velocities stay approximately the same as they propagate in the front.

Supplementary Figures 4 (c), and (d) present the calculated average power of the wave packets and their trajectories for the case of $\Delta\omega_{shift}$ being sufficient to induce intraband transitions (the same as Fig. 5 in the main text but without correcting the time by the slope of the pump group velocity). Now, from Supplementary Figure 4 (c) we can observe that the energies of the wave packets are shifted and occur at shorter wavelengths, as observed in our experiment. Compared to the previous case, the wave packets got accelerated after interacting with the front, as shown in Supplementary Figure 4 (d).

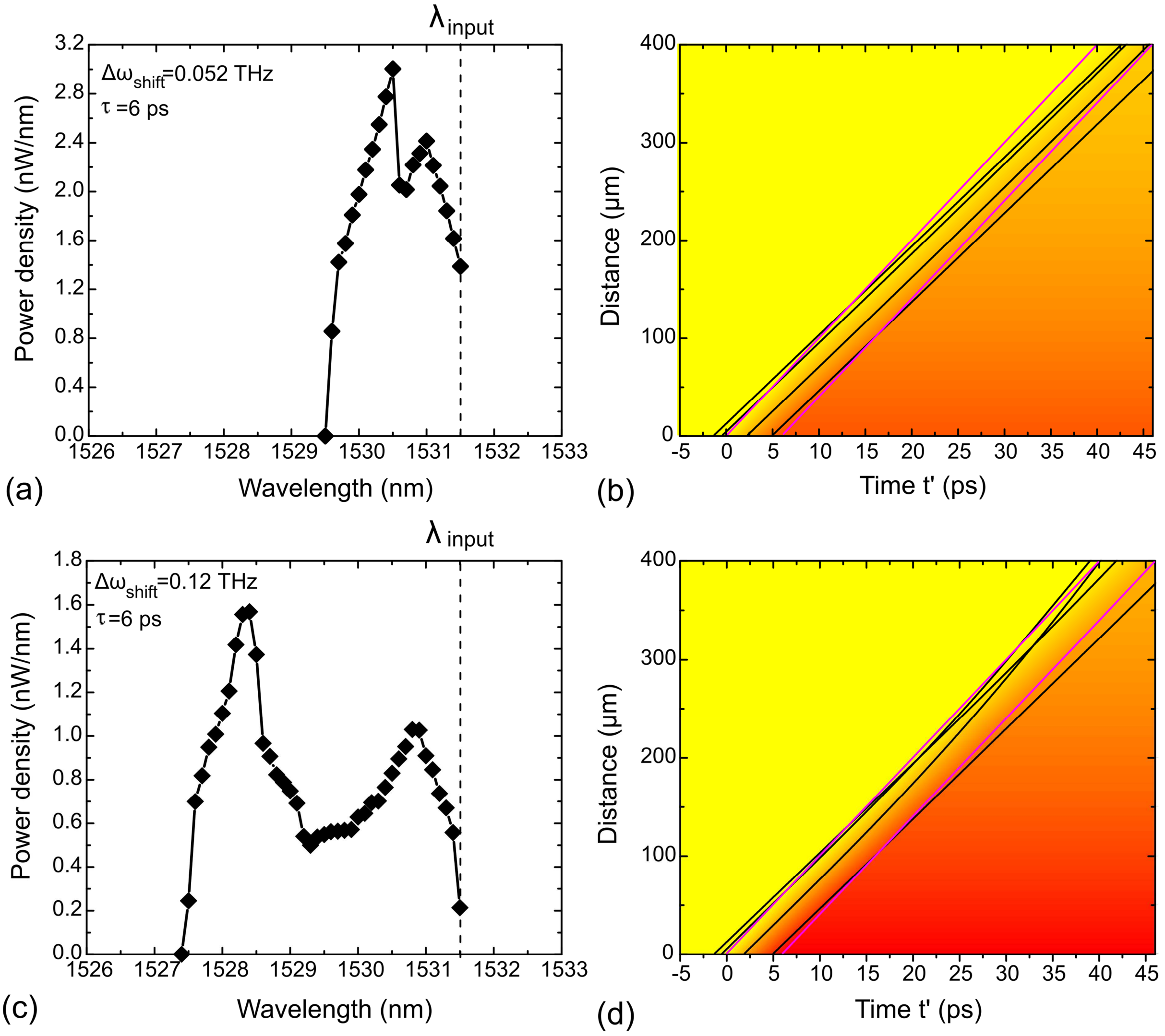


Supplementary Figure 4: Simulation of the FC front interaction with CW probe wave packets in case of (a, b) $\Delta\omega_{shift}$ is insufficient to induce intraband transition, and (c, d) $\Delta\omega_{shift}$ is sufficient to induce intraband transition. Left: Average power spectrum of the frequency shifted wave packets at 6 μW output CW probe power at 1531.5 nm, rising time of the front of 6 ps, Right: their trajectories. The two magenta lines represent the index front with a slope equal to the group velocity of the pump and rising time of 6 ps. The shading represents the front refractive index changes exponentially decaying during propagation inside the waveguide. Black lines represent those wave packets that interacting with the front and undergo both interband and intraband transitions.

Supplementary Figures 5 (a), and (b) present the calculated average power of the wave packets interacting with 1 ps long pump pulse -with the same input pump peak power- and their trajectories for the case of $\Delta\omega_{shift}$ being sufficient to induce intraband transitions, respectively. In this case the time spent by the wave packets inside the front Δt is shorter than in case of 6 ps long pump, therefore the wave packets will experience less FC absorption

before reflection. In turn, the reflection efficiency will be increased compared to 6 ps long pulses. Recalling equation 2 in the main manuscript, the maximally transformable fraction of the incoming CW probe power in this case due to the interaction with single pump pulses arriving at 100 MHz repetition rate is $0.5\cdot10^{-3}$. Thus the maximal average power of 3 nW would be expected at output for transmitted 6 µW CW probe. We obtain 2.1 nW average power of the shifted light, which corresponds to 70% conversion efficiency. Compared to the case of 6 ps pump, here the reflection efficiency will be 2 times higher.

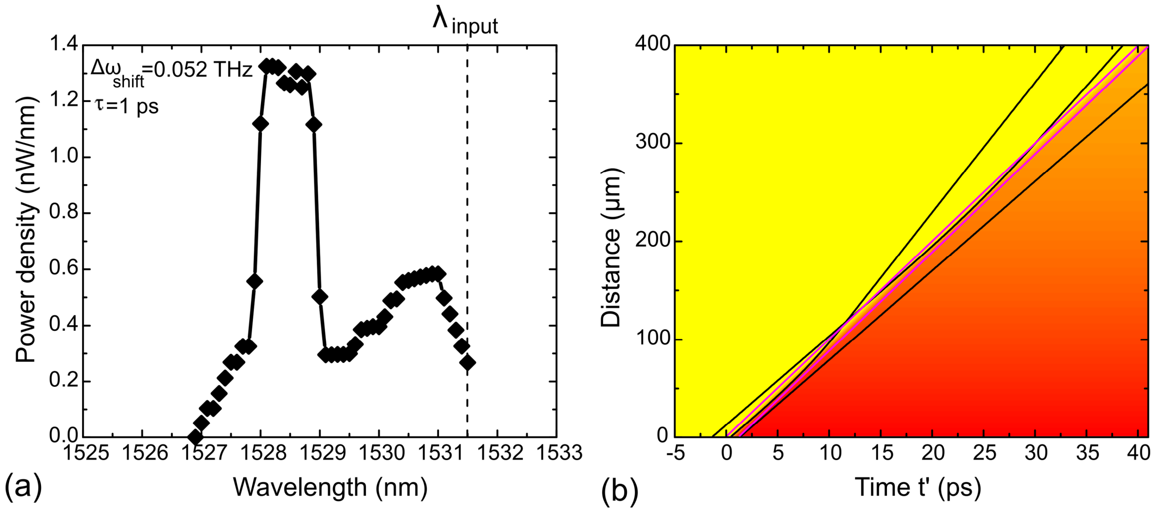


Supplementary Figure 5: Simulation of the FC front interaction with CW probe wave packets in case of $\Delta\omega_{shift}$ is sufficient to induce an intraband transition with a pump pulse duration of 1 ps with the same pump peak power as for the case of 6 ps. Left: average power spectrum of the frequency shifted wave packets at the output, Right: their trajectories. The output CW probe power is 6 µW at 1531.5 nm.

## Supplementary References


1. Li, J., White, T. P., O'Faolain, L., Gomez-Iglesias, A. & Krauss, T. F. Systematic design of flat band slow light in photonic crystal waveguides. Opt. Express 16, 6227–6232 (2008).
2. Li, J., O'Faolain, L., Rey, I. H. & Krauss, T. F. Four-wave mixing in photonic crystal waveguides: slow light enhancement and limitations. Opt. Express 19, 4458–4463 (2011).
3. Gomez-Iglesias, A., O'Brien, D., O'Faolain, L., Miller, A. & Krauss, T. F. Direct measurement of the group index of photonic crystal waveguides via Fourier transform spectral interferometry. Appl. Phys. Lett. 90, 261107 (2007).

4. Dekker, R. et al. Ultrafast Kerr-induced all-optical wavelength conversion in silicon waveguides using 1.55 μm femtosecond pulses. Opt. Express 14, 8336–8346 (2006).
5. Turner-Foster, A. C. et al. Ultrashort free-carrier lifetime in low-loss silicon nanowaveguides. Opt. Express 18, 3582–3591 (2010).
6. Soref, R. & Bennett, B. Electrooptical effects in silicon. IEEE Journal of Quantum Electronics 23, 123–129 (1987).
7. J. D. Joannopoulos, S. G. Johnson , J. N. Winn & R. D. Meade. Photonic Crystals, Molding the Flow of Light (Princeton University Press, 17–19 (2008), Princeton, NJ).
8. Munoz, M. C., Kanchana, A., Petrov, A. Y. & Eich, M. Dynamic Light Storage in Slow Light Waveguides. IEEE Journal of Quantum Electronics 48, 862–866 (2012).
9. O'Faolain, L. et al. Loss engineered slow light waveguides. Opt. Express 18, 27627–27638 (2010).
10. Zhang, Y. et al. Non-degenerate two-photon absorption in silicon waveguides: analytical and experimental study. Opt. Express 23, 17101–17110 (2015).
11. Schroder, D. K., Thomas, R. N. & Swartz, J. C. Free Carrier Absorption in Silicon. IEEE Journal of Solid-State Circuits 13, 180–187 (1978).
